# SARS-CoV-2 Infection in Winter 2021/2022: The Association of Varying Clinical Manifestations With and Without Prior Vaccination

**DOI:** 10.7759/cureus.33190

**Published:** 2022-12-31

**Authors:** Menachem Jacobs, Avrohom Karp, Christopher Alessandro, Marc Ganz, Aaron Glatt, Avi Rosenberg, Ruoyu Wang, Jason Zimmerman, Jonathan Silverberg, Israel Zyskind

**Affiliations:** 1 Public Health Sciences, State University of New York Downstate Health Sciences University, Brooklyn, USA; 2 Health Sciences, New York Medical College, Valhalla, USA; 3 Infectious Disease, Mount Sinai Medical Center, Queens, USA; 4 Pathology and Laboratory Medicine, Johns Hopkins University, Baltimore, USA; 5 Medicine, State University of New York Downstate Health Sciences University, Brooklyn, USA; 6 Emergency Medicine, Maimonides Medical Center, Brooklyn, USA; 7 Dermatology, George Washington University, Washington, D.C., USA; 8 Pediatrics, New York University (NYU) Langone, Brooklyn, USA

**Keywords:** covid-19, sars-cov-2 immunity, booster vaccine, covid-19 vaccination, latent class analysis

## Abstract

Importance

SARS-CoV-2 is a rapidly evolving virus with many strains. Although vaccines have proven to be effective against earlier strains of the virus, the efficacy of vaccination status against later strains is still an area of active research.

Objective

To determine if vaccination status was associated with symptomatology due to infection by later strains of SARS-CoV-2.

Design

This cross-sectional survey was sent to an adult Jewish population from December 2021 to March 2022.

Setting

This is a population-based study of Jewish communities throughout the tristate area. The subjects were recruited by local Jewish not-for-profit and social service organizations.

Participants

Surveys were sent to 14,714 adults who were recruited by local Jewish not-for-profit and social service organizations; 966 respondents completed the survey (6.57%). Only participants who received a positive COVID-19 nasal swab 10 weeks since December 1, 2021, were included in the main outcome.

Exposure

Participants were grouped by vaccine type (i.e., Johnson & Johnson {J&J}, Moderna, or Pfizer) and vaccination status (i.e., unvaccinated, single, full, or booster).

Main outcomes and measures

The primary study outcome was an association between immunization status and somatological presentation. Symptom severity classes were built using latent class analysis (LCA).

Results

Out of 14,714 recipients, 966 completed the survey (6.57%). The participants were mainly self-described Ashkenazi Jewish (97%) with a median age of 41. The LCA resulted in four classes: highly symptomatic (HS), less symptomatic (LS), anosmia, and asymptomatic (AS). Vaccinated participants were less likely to be in symptomatic groups than the unvaccinated participants (odds ratio {OR}: 0.326; 95% confidence interval {CI}: 0.157-0.679; p=0.002). Boosted participants were less likely to be in symptomatic groups than fully vaccinated participants (OR: 0.267; 95% CI: 0.122-0.626; p=0.002). Additionally, there was no association between symptomatology and vaccination type (p=0.353).

Conclusions and relevance

Participants who received COVID-19 vaccinations or booster shots were less likely to be symptomatic after Omicron infection compared to unvaccinated participants and vaccinated participants without boosters, respectively. There’s no association between vaccination type and symptomatology. These results enhance our understanding that COVID-19 vaccinations improve clinical symptomatology, even in an unforeseen COVID-19 strain.

## Introduction

By the end of 2021 and the beginning of 2022, several new Omicron strains of the SARS-CoV-2 virus rose to prominence and quickly replaced the prior predominant Delta variant as the major strain in the United States. SARS-CoV-2 Omicron variant, subsequently referred to as BA.1, was one such strain and was first identified in South Africa on November 24, 2021. This variant has proven to be more contagious than previous strains, with infection reported in all six World Health Organization regions and 149 countries within a few weeks [1,2].

Omicron spread rapidly in the United States, with the first cases reported on December 1, 2021 [2,3]. By January 1, 2022, 95% of SARS-CoV-2 cases were attributed to the Omicron BA.1 variant. Since then, newer Omicron strains have replaced the BA.1 variant, with the BA.5 variant comprising over 88.8% of the US strains as of August 12, 2022 (CDC data accessed on August 12, 2022) [4].

Much of the transmissibility of Omicron has been attributed to differences in the genetic code found in the earliest days of Omicron sequencing, which discovered >30 mutations in the spike protein and receptor domains [5,6]. This led to concern about whether these mutations would alter the virus’s transmissibility or lead to immune escape. Immune escape describes the virus’s ability to evolve and infect previously immune individuals. It was seen even in areas that were previously shielded due to higher vaccination rates or previous infection with the SARS-CoV-2 virus [7-11].

Vaccines for SARS-CoV-2 have proven to be highly effective at preventing severe disease and fatalities in the original and Alpha strains of COVID-19 [12-17]. However, vaccine efficacy has been more modest in preventing symptomatic disease with the subsequent Beta and Delta variants, even among those who received the recommended two-dose vaccine regimen [18-21]. The SARS-CoV-2 B.1.1.529 Omicron variant showed the steepest reduction in vaccine efficacy provided by vaccination. A reduction in immunity was witnessed where the initial 65% immunity acquired after two doses of the BNT162b2 Pfizer vaccine at 2-4 weeks was reduced to a mere 8.8% at 25 weeks and onward, according to various findings [22].

In response to the waning protection granted by vaccination [23], the CDC recommended that previously doubly vaccinated patients receive an additional dose of an mRNA vaccine, referred to in the vernacular as “boosting” [24], to enhance the levels of protection against breakthrough infection. The administration of boosters resulted in renewed protection against mild infection, but as with the original vaccinations, the protective effects diminished over time [25].

While the rates of COVID-19 breakthrough in previously vaccinated and boosted individuals have been studied [26], data describing the utility of vaccination to mitigate severe COVID-19 infection and symptomatology are still sparse, and how the severity of symptoms relates to differing vaccination status is still an important area of research.

Survey data were analyzed to determine the relationship between clinical and symptomological manifestations of late 2021 or early 2022 COVID-19 variants with a prior vaccination history and/or previous COVID-19 infection. The phenotypes of symptomatology and their relationship with previous COVID-19 diagnoses and varying vaccination status are described.

Given the data regarding vaccination- and booster-induced immunity, this study aimed to determine if vaccination and booster use correlated with reduced symptoms within the cohort, as measured by their membership in various groups in a latent class analysis (LCA). The LCA is an interesting and impressive machine learning modality with regard to COVID-19 and can be a useful tool in parsing symptomatology in data gathered from self-reporting.

## Materials and methods

Study design

The subjects were recruited by local not-for-profit and social service organizations within Orthodox Jewish communities throughout the tristate area. A cross-sectional survey invitation was sent to 14,714 adults, of whom 1,020 individuals began the survey process (6.93% response rate). The number of participants to complete the survey was 966 (of 1,020, 94.7%). This was the third survey sent to this cohort over the pandemic, which possibly explains the low response rate. Electronic informed consent was taken, and the study’s purpose was disclosed before beginning the survey. The study was open to all participants and did not require participants to have SARS-CoV-2 symptoms or exposures to participate.

The survey was developed to determine the most common symptoms associated with infections later in the pandemic and examine the relationship between clinical outcomes in a community-based cohort with previous vaccination and infection between December 1, 2020, and March 1, 2021. The survey included 25 data points, including questions about patient demographics, symptoms of infection, and whether they tested positive for SARS-CoV-2 by nasal swab. The survey was administered via the Health Insurance Portability and Accountability Act-compliant and secure Research Data Capture (REDCap) (Vanderbilt University, Nashville, TN) software. The Advarra Institutional Review Board approved the study (approval number: MOD01212191).

Data analysis

Baseline characteristics were determined, and summary statistics were estimated. We charted the symptoms of those respondents that tested positive and grouped them into four classes using latent class analysis (LCA) based on common clinical presentations. Twenty-four distinct symptoms were assessed, and 20 were ultimately included based on the criteria outlined by Miaskowski et al. where a minimum of five participants (2%) was required to exhibit a symptom for the symptom to be included in the analysis establishing a cutoff [27]. ​​A latent class analysis (LCA) was then used to examine the phenotypic patterns of COVID-19 symptoms.

LCA uses observed categorical or binary data to identify patterns known as latent classes. We used conditional probabilities to estimate the likelihood that a member of the survey cohort belonged to a group based on their response to specific symptoms of COVID-19, thereby allowing the characterization of latent classes. Pearson’s chi-square analysis was used to demonstrate frequency differences regarding prior infection status and the type of vaccine received. Subsequently, odds ratios (ORs) were calculated to compare each class individually.

All data processing and statistical analyses were performed in Statistical Analysis System (SAS) (SAS Institute Inc., Cary, NC) version 9.4.3 and Statistical Package for Social Sciences (SPSS) (IBM SPSS Statistics, Armonk, NY) version 28. Complete data analysis was performed; the subjects with missing data were excluded. A two-sided p of less than 0.05 was considered statistically significant.

## Results

Population characteristics

The survey cohort had an interquartile age of 41 and was composed of 54% males and 46% females. The patient population was overwhelmingly self-described Ashkenazi Jewish (97%). Among the 966 sufficiently filled respondents in the survey cohort, 217 patients reported SARS-CoV-2 symptoms, with 229 (24%) reporting a positive nasal swab test within the past 10 weeks (since December 1, 2021). The most commonly reported symptoms were fatigue (64.7%), followed by cough (53.1%), sore throat (46.9%), and aches (45.1%). The symptoms persisted for an average of 5.29 days (SD: 3.41).

Of the 966 respondents, 609 (63.0%) received at least one vaccination. Of the 609 vaccinated participants, 584 (95.9%) received their “full” (two-dose mRNA) vaccination series, and 246 (42.1%) of these 584 fully vaccinated respondents received “booster” doses of mRNA vaccine.

We charted the symptoms of those respondents that tested positive and grouped them into four classes using latent class analysis (LCA) based on the typology of symptoms (Table 1). Twenty-five distinct symptoms were assessed, of which 19 were ultimately deemed to be significant. Four distinct classes of symptomatology were identified. Based on the symptomatic presentation, these classes were labeled as class 2, highly symptomatic (HS); class 3, less symptomatic (LS); class 1, anosmia; and class 4, asymptomatic (AS).

**Table 1:** Frequency of Vaccinated, Unvaccinated, and Boosted Participants in LCA Groupings LS, Less Symptomatic; HS, Highly Symptomatic; LCA, Latent Class Analysis

Class: Vaccinated Versus Unvaccinated Versus Boosted	Number of Participants	Unvaccinated	Vaccinated and Unboosted	Vaccinated and Boosted
Class 3: LS	126 (58.1%)	58	51	17
Class 4: Asymptomatic	53 (24.4%)	11	21	21
Class 2: HS	27 (12.4%)	8	15	4
Class 1: Anosmia	11 (5.1%)	7	4	0
Total	217	84	91	42

HS (class 2) presented with upper respiratory infection (URI)-like symptoms: fatigue (99.6%), aches (95.5%), cough (82%), and headache (75.7%). LS (class 3) was primarily defined by fatigue as its most prominent characteristic (81.3%), although it was also associated with weakness (57%), cough (55%), and aches (54%). The asymptomatic class (class 4) included patients who were mildly symptomatic, with the primary symptoms consisting of cough (39%), sore throat (39%), and other tertiary symptoms (23.8%). Finally, the anosmia class (class 1) consisted of patients with symptoms most similar to mild Alpha strain infection, with fatigue (90.5%), weakness (89.1%), the loss of smell (82.9%), and headache (58%) being the most common symptoms in this class.

Vaccination status and severity of symptoms

Overall, the four classes were associated with different frequencies of vaccination status (p=0.003). The vaccinated participants were less likely to be symptomatic than the unvaccinated participants (OR: 0.326; 95% CI: 0.157-0.679; p=0.002). They were also much less likely to belong to the anosmia class than the asymptomatic class (OR: 6.682; 95% CI: 1.65-26.99; p=0.008). They were less likely to belong to class 3 (LS) than asymptomatic (OR: 3.257; 95% CI: 1.537-6.899; p=0.002) (Table 2).

**Table 2:** Vaccinated Versus Unvaccinated OR OR, Odds Ratio; CI, Confidence Interval; LS, Less Symptomatic; HS, Highly Symptomatic

Vaccinated Versus Unvaccinated	OR	95% CI	P-Value
Symptomatic Versus Asymptomatic (AS)	0.326	0.157-0.679	0.002
Class 1 (Anosmia) Versus Class 4 (AS)	6.682	1.65-26.99	0.008
Class 3 (LS) Versus Class 4 (AS)	3.257	1.537-6.899	0.002
Class 1 (Anosmia) Versus Class 2 (HS)	4.156	0.946-18.265	0.073
Class 1 (Anosmia) Versus Class 3 (LS)	2.052	0.572-7.360	0.350
Class 2 (HS) Versus Class 3 (LS)	0.494	0.201-1.211	0.138
Class 2 (HS) Versus Class 4 (AS)	1.608	0.557-4.639	0.413

Additionally, we conducted a chi-square test to determine associations between symptomatology and vaccination type (Pfizer, Moderna, and Johnson & Johnson {J&J} series) and found no statistical difference (p=0.353).

Boosted versus vaccinated

Of the doubly vaccinated respondents, 48.3% received a “booster” dose of the mRNA vaccine (Table 3). The symptomatic class was less likely to have been boosted compared to the asymptomatic class (OR: 0.267; 95% CI: 0.122-0.626; p=0.002). Class 1 (anosmia) was less likely to have been boosted than class 4 (AS) (OR: 2.313; 95% CI: 1.599-3.345; p=0.048). Class 3 (LS) was less likely to have been boosted compared to class 4 (AS) (OR: 3.243; 95% CI: 1.371-7.667; p=0.010). Class 2 (HS) was less likely to have been boosted compared to class 4 (AS) (OR: 3.938; 95% CI: 1.068-14.523; p=0.041) (Table 4).

**Table 3:** Boosted Versus Vaccinated Frequency HS, Highly Symptomatic; LS, Less Symptomatic

Class: Boosted Versus Vaccinated	Number of Participants	Boosted	Unboosted
Class 2: HS	16 (13.8%)	4	12
Class 3: LS	59 (50.9%)	17	42
Class 4: Asymptomatic	37 (31.9%)	21	16
Class 1: Anosmia	4 (3.4%)	4	0

**Table 4:** Boosted Versus Vaccinated Odds Ratio OR, Odds Ratio; CI, Confidence Interval; LS, Less Symptomatic; HS, Highly Symptomatic

Boosted Versus Vaccinated	OR	95% CI	P-Value
Symptomatic Versus Asymptomatic (AS)	0.276	0.122-0.626	0.002
Class 1 (Anosmia) Versus Class 4 (AS)	2.313	1.599-3.345	0.048
Class 3 (LS) Versus Class 4 (AS)	3.243	1.371-7.667	0.010
Class 2 (HS) Versus Class 4 (AS)	3.938	1.068-14.523	0.041
Class 1 (Anosmia) Versus Class 2 (HS)	1.333	1.005-1.769	0.538
Class 1 (Anosmia) Versus Class 3 (LS)	1.405	1.194-1.652	0.330
Class 2 (HS) Versus Class 3 (LS)	1.214	0.343-4.298	1

Boosted versus unvaccinated

Furthermore, we conducted an LCA analysis comparing boosted respondents with non-vaccinated respondents (Table 5). The lesser symptomatic and asymptomatic classes had a higher proportion of boosted participants as compared to the unvaccinated participants.

**Table 5:** Boosted Versus Unvaccinated OR OR, Odds Ratio; CI, Confidence Interval; LS, Less Symptomatic; HS, Highly Symptomatic

Boosted Versus Unvaccinated	OR	95% CI	P-Value
Class 1 (Anosmia) Versus Class 2 (HS)	1.500	1.005-2.238	0.245
Class 1 (Anosmia) Versus Class 3 (LS)	1.293	1.144-1.462	0.335
Class 1 (Anosmia) Versus Class 4 (AS)	2.909	1.802-4.695	0.002
Class 2 (HS) Versus Class 3 (LS)	0.586	0.157-2.186	0.473
Class 2 (HS) Versus Class 4 (AS)	3.818	0.937-15.554	0.088
Class 3 (LS) Versus Class 4 (AS)	6.513	2.627-16.148	<0.001
Symptomatic Versus Asymptomatic	6.636	2.763-15.939	<0.001

Class 1 (anosmia) was significantly less likely to have gotten their booster than those in class 4 (AS) (OR: 2.909; 95% CI: 1.802-4.695; p=0.002). Class 3 (LS) was significantly less likely to have gotten their booster than those in class 4 (AS) (OR: 6.513; 95% CI: 2.627-16.148; p<0.001). Symptomatic respondents were significantly less likely to have gotten their booster than those in asymptomatic (OR: 6.636; 95% CI: 2.763-15.939; p<0.001).

## Discussion

By the winter of 2021, breakthrough infections were confirmed in previously vaccinated individuals [23]. While the rates of COVID-19 breakthrough in previously vaccinated and boosted individuals have been studied [25], the relationship between the symptomatology of COVID-19 breakthrough infection with prior vaccination and boosting status is less understood.

We administered a survey asking patients to report their vaccination history, incidence of breakthrough infection, and resulting symptomatology from December 2021 to March 2022. We aimed to discern differences in the symptomatology of confirmed COVID-19 cases among patients who received single, full, or booster vaccination status. We conducted an LCA analysis, grouping individuals into four discrete categories based on their reported symptoms. We found that there was a protective effect conferred by the vaccine and booster vaccination, as demonstrated by a reduction in COVID-19 symptomatology as reported in the survey. We found that vaccination (OR: 0.326; 95% CI: 0.157-0.679; p=0.002) and especially boosting in previously vaccinated individuals (OR: 0.267; 95% CI: 0.122-0.626; p=0.002) were associated with clinically less severe COVID-19 symptomatology groupings based on their self-reported COVID-19 symptoms.

Our survey cohort had a mix of boosted vaccinated (42.1%) and non-boosted but vaccinated participants (57.9%). The completion of the primary two-dose mRNA vaccination series and the provision of a third dose, “boosting,” decreased the likelihood of contracting highly symptomatic reinfection of SARS-CoV-2 in our cohort, as defined by increased membership to the previously described highly symptomatic group as opposed to the asymptomatic group.

The use of LCA in this context is an interesting machine learning modality with regard to COVID-19 and can be a useful tool in parsing symptomatology in data garnered from self-reporting [26].

Our study has limitations. Our population was limited in scope, with the vast majority of respondents sharing socio-economic and cultural similarities and coming from an ethnically homogenous Ashkenazi Jewish community. Additionally, age was not well distributed in our cohort, with 344 people (36%) of our participants between 40 and 60 years of age.

## Conclusions

There has been much doubt among the general population regarding the efficacy of COVID-19 vaccination and booster vaccines. The data presented here highlight the protective effects conferred by vaccinating and receiving a booster vaccine on the overall symptoms of individuals infected by COVID-19. This may then be used to provide a more robust understanding of the benefits of receiving vaccinations and boosters in the general public.
